# Congenital esophageal stenosis diagnosed in an infant at 9 month of age

**DOI:** 10.1186/s13052-015-0182-y

**Published:** 2015-10-06

**Authors:** F. Savino, V. Tarasco, S. Viola, E. Locatelli, M. Sorrenti, A. Barabino

**Affiliations:** Department of Pediatrics, Regina Margherita Children’s Hospital, University of Torino, Città della Salute e della Scienza di Torino, Torino, Italy; Gastroenterology and Endoscopy Unit - G. Gaslini Institute for Children, Genova, Italy

**Keywords:** Dysphagia, Congenital esophageal stenosis, Infant, Endoscopic balloon dilatation

## Abstract

Esophageal stenosis is a relatively uncommon condition in pediatrics and requires an accurate diagnostic approach. Here we report the case of a 9-month old female infant who presented intermittent vomiting, dysphagia and refusal of solid foods starting after weaning. She was treated for gastroesophageal reflux. At first, radiological investigation suggested achalasia, while esophagoscopy revelaed a severe congenital esophageal stenosis at the distal third of the esophagus. She underwent four endoscopic balloon dilatations that then allowed her to swallow solid food with intermittent mild dysphagia. After 17 months of esomeprazole treatment off therapy impedance-pH monitoring was normal. At 29 months of follow-up the child is asymptomatic and eats without problems.

Infants with dysphagia and refusal of solid foods may have undiagnosed medical conditions that need treatment. Many disorders can cause esophageal luminal stricture; in the pediatric age the most common are peptic or congenital. Careful assessment with endoscopy is needed to diagnose these conditions early and referral to a pediatric gastroenterologic unit may be necessary.

## Introduction

Vomiting in early infancy commonly occurs and most frequently is diagnosed as gastroesophageal reflux. Atypical presentations can present a diagnostic challenge, particularly after the neonatal period. Esophageal impaction with refusal of solid foods in infants is a medical problem that needs immediate medical attention [1].

Esophageal stenosis is a relatively uncommon condition in pediatrics but must be considered in differential diagnosis. Stenosis in children is often due to the ingestion of alkali and acid corrosive substances, or accidental button battery ingestion [2–4].

In the last years multiple bits and pieces (toys, food, coins and other) have been detected and removed endoscopically [5]. Also, eosinophilic esophagitis has been associated with esophageal stenosis, leading to esophageal impaction [4].

In the current article, we present our experience in an infant who presented intermittent vomiting, dysphagia and refusal of solid foods starting after weaning and referred to our Pediatrics service.

## Case report

The infant was born at term by spontaneous delivery after an uncomplicated pregnancy; birth weight was 2810 g and APGAR score was nine. Perinatal history was unremarkable. She was exclusively breastfed until six months of life with appropriate growth and neurological development and no clinically relevant feeding difficulties. When weaning was started and semisolid meals were introduced, she started to present some problems like refusal of food and vomiting. Parents reported no taste selectivity or difficulties with ingestion of liquid and homogenized food. For these reasons and because of the failure to thrive the infant was taken to the emergency department for a first time and discharged after 3 days of hospitalization with a diagnosis of gastroenteritis. An abdominal ultrasound was performed and revealed no pathological images. Because of the persistent food refusal, vomiting and loss of weight the infant was referred to our tertiary pediatric care centre at 9 months of age.

**Fig. 1:**
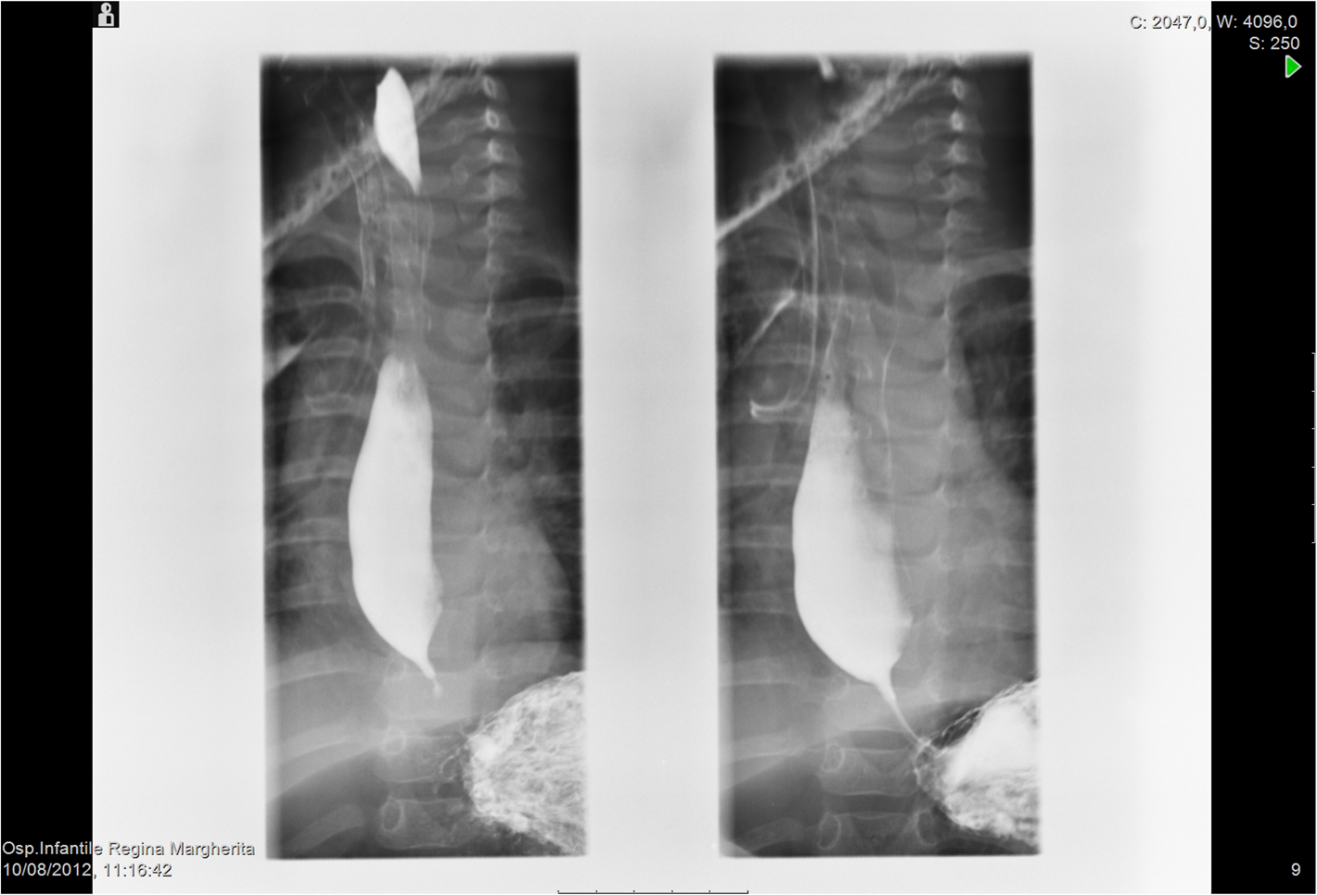
Fluoroscopy with barium: Image of esophageal suggestive of achalasia in an infant at 9 months of age. Fluoroscopic images from a barium esophagogram reveal a persistently dilated esophagus with an air-fluid level at the lower esophagus and classic "bird-beaking" at the gastroesophageal junction, and normal primary peristaltic waves

**Fig. 2:**
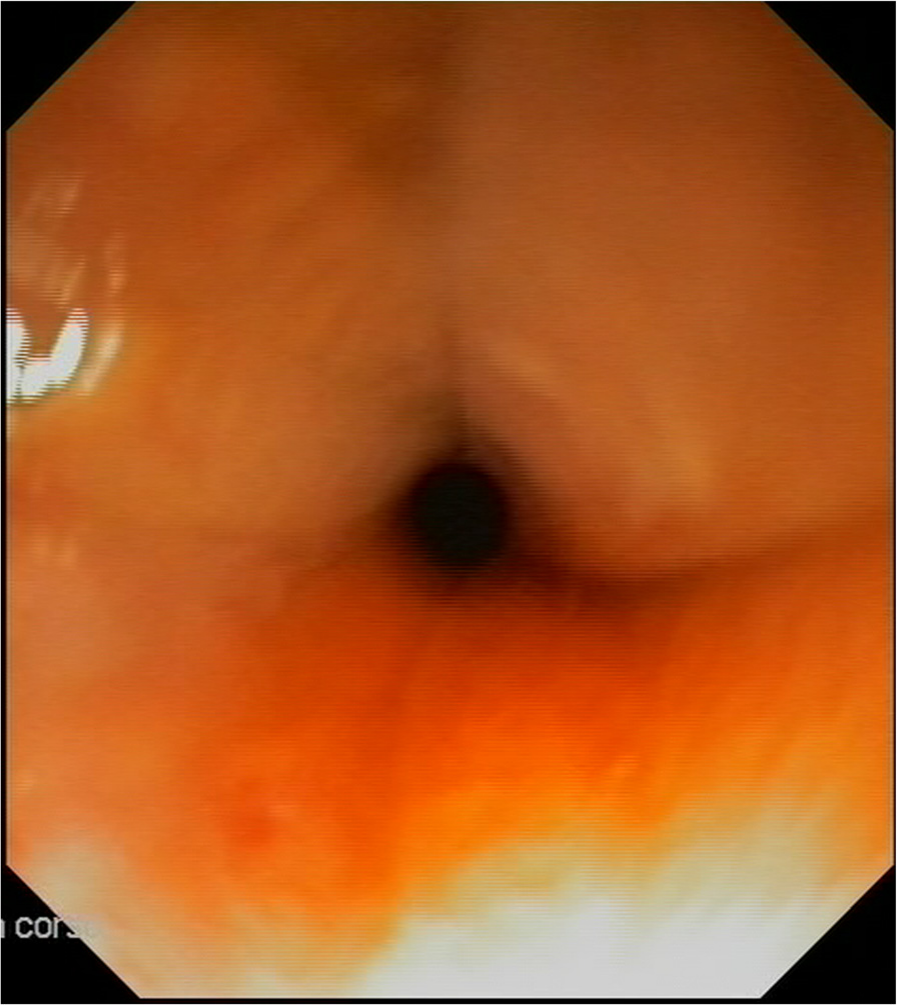
Endoscopy showing esophageal stenosis. Endoscopic view just few centimeters above the cardias, within a normal mucosa, a firm stenosis did not allow the passage of the endoscope (5.6 mm diameter). This finding was not compatible with achalasia, and it suggested the diagnosis of esophageal stenosis

On admission she presented good general condition. Physical examination was unremarkable with a weight of 7460 g (<10 th centile). Haemochrome, renal and hepatic function were within normal limits; urine and stool analysis were normal. An observational feeding trial showed signs of dysphagia: the infant repeatedly aborted weaning meals after a few minutes followed by crying, gagging and vomiting. Therefore a diagnostic process was started to evaluate the possible different causes of dysphagia. A tracheoscopy was performed and showed no visual abnormalities in the pharynx and larynx. Neurological evaluation was normal. The esophageal radiographic studies suggested achalasia (Fig. 1). An esophagogram showed tapered narrowing at the distal third of the esophagus with slightly suprastenotic dilatation. To check this diagnosis an esophagoscopy was performed (Videogastroscope XP180, Olympus, Milan, Italy) (Fig. 2). Just a few centimeters above the cardias, within a normal mucosa, a firm stenosis did not allow the passage of the endoscope (5.6 mm diameter). The approximate diameter of the esophageal lumen in the stenotic tract was 2–3 mm and its length about 5 mm. This finding was not compatible with achalasia, and suggested the diagnosis of congenital esophageal stenosis.

**Fig. 3:**
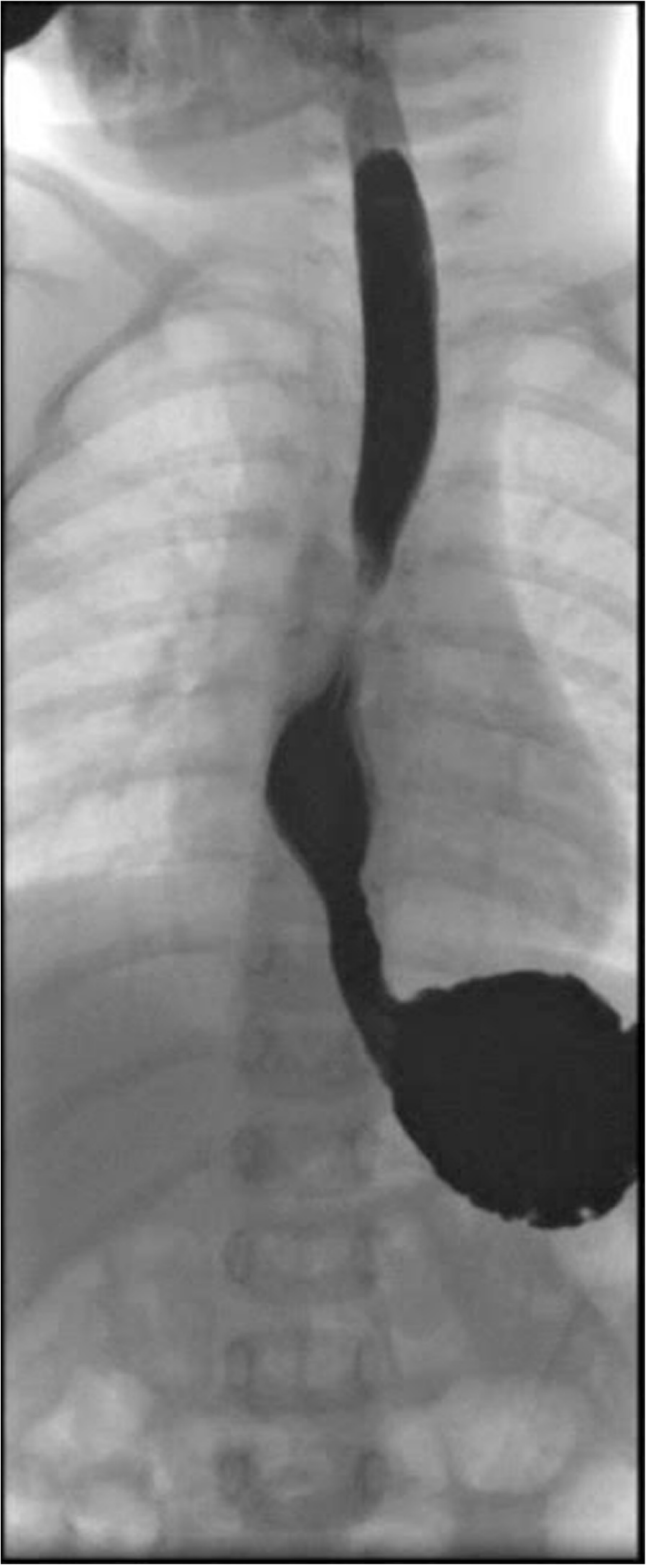
Fluoroscopy with barium: Image of esophagus in the infant 17 months after the last operative endoscopy: a slight dilatation of the esophagus above the site of the previous stenosis

Echoendoscopy, carried out by means of a through the scope mini-probe (20 MHtz, Olympus, Milan, Italy), showed a regular thickening of the esophageal wall in the absence of cartilagenous remnants. After, high pressure catheter (CRE Wireguided, Boston Scientific, Cork, Ireland) was passed over the guide wire and positioned across the stricture, and slowly inflated using radiopaque contrast medium. The balloon was considered to be correctly positioned when the “waist” of the balloon catether was in the center of the balloon. Inflation lasting up to 1 min, was performed manually until the disappearance of the waist. After the first balloon dilatation from 6 to 8 mm, a normal stomach was reached. Afterwards every 15 days the girl underwent three fluoroscopically guided balloon dilatations without complications. The balloon diameter was slowly increased from 8 to 12 mm, that allowed the final passage of an endoscope of 8.6 mm diameter (Videogastroscope GIF 180, Olympys, Milan, Italy). After each dilatation a 3 day-course of oral betametasone was given (dosage 0.1 mg per kilo per day). Taking into account the site of the stenosis and fearing a possible subsequent gastro-esophageal reflux, after the first dilatation a prolonged treatment with proton pump inhibitor (PPI), esomeprazole at a dosage of 1 mg per kilo per day was started (6). At the end of the dilatation program the child was able to swallow solid foods with intermittent mild dysphagia. An esophagogram done 5 and 17 months (Fig. 3) after the last operative endoscopy showed a slight dilatation above the site of the previous stenosis. After 17 months of esomeprazole treatment off therapy impedance-pH monitoring resulted normal and the drug was definitively stopped. At 29 months of follow-up the child is asymptomatic and she is able to eat without problems.

### Consent to publish

Written informed consent was obtained from parents of the patient for publication of this Case report and any accompanying images. A copy of the written consent is available for review by the Editor-in-Chief of this journal.

## Discussion

In the present article, we report our experience with a 9-month old female infant who presented intermittent vomiting, dysphagia and refusal of solid foods starting after weaning with congenital esophageal stenosis.

Although a number of studies have documented the epidemiology and potential etiologies of esophageal food impaction (EFI) in children [3], few reports have documented this issue in infants less than one year of age [2].

Feeding and swallowing disorders in children (pediatric dysphagia) are considered a major challenge owing to a wide differential diagnosis [1–4].

The causes of dysphagia are arguably varied and include combinations of structural deficits, neurologic conditions, respiratory compromise, feeder–child interaction dysfunction, and numerous medical conditions including genetic, metabolic, and degenerative diseases [1–4]. In particular, it is commonly associated with obstructive or motor disorders of the esophagus. Indeed, the oropharyngeal dysphagia, in which solids and liquids cannot move out of the mouth properly, could be related to psychological problems [2, 3]. Patients with obstructive disorders such as esophageal stenosis are unable to swallow solids but can tolerate liquids; this condition is often called EFI. Many cases of EFI in children are caused by an esophageal foreign body, causing acute dysphagia, and meat is the most common food impacted [3].

Among anatomic abnormalities, esophageal stricture is reported to be a rare but possible cause of dysphagia. It is defined as a fixed, intrinsic narrowing of the esophagus that obstructs the normal aboral propagation of a swallowed bolus. Esophageal stenosis can be congenital (CES) or acquired. The definition and classification of CES proposed by Nihoul-Fékété et al. are the clearest. They state that CES is defined as an intrinsic stenosis of the esophagus, present at birth, and associated with congenital malformation of the esophageal wall architecture. CES is categorized in 3 types: fibromuscular stenosis (FMS), esophageal membranes or web and tracheobronchial remnants (TBR). The acquired stenosis can be divided into the following categories: traumatic, inflammatory, peptic, and after surgery [4, 5]. Congenital esophageal stenosis is rarely diagnosed in neonates because the onset of symptoms usually begins with the introduction of solid food at the age of 4 to 10 months. A history of dysphagia to solid food and a lack of weight gain are important in establishing a suspicion of CES [6].

In our case, the infant exclusively tolerated breastfeeding and she started to present feeding problems, in particular refusal of food and vomiting, when semisolid meals were introduced during weaning. This data gave the suspicion of an obstructive disorder probably due to fibromuscular stenosis .

The first diagnostic step was to exclude the oropharyngeal causes of dysphagia. Therefore, we performed otorhinolaryngoiatric and neurologic evaluations. In our patient tracheoscopy did not show any visual abnormalities in the pharynx and larynx and neurological evaluation resulted normal.

The radiologic study is instead considered the first step to recognize an obstructive esophageal disorder [7]. In our patient it resulted compatible with the diagnosis of achalasia, which is defined as an esophageal motility disorder involving the smooth muscle layer of the esophagus and the lower esophageal sphincter [8]. Literature reports have demonstrated that both symptoms and radiological features of achalasia can be similar to the ones of more common disorders, such as gastroesophageal reflux disease, or CES. The differential diagnosis can be performed with esophageal endoscopy: the endoscope cannot be forced through the stenosis as in achalasia [7]. In CES, endoscopy typically shows a concentric narrowing without mucosal alterations and, in particular, no macroscopic or histologic alterations owing to reflux esophagitis or eosinophilic esophagitis. This investigation let us exclude the diagnosis of achalasia and it documented the presence of an esophageal stenosis.

CES is a rare malformation with an incidence of approximately 1 in 25,000 to 50,000 live births [9, 10]: however we have to consider the possibility of this diagnosis in infants presenting with dysphagia.

Surgery, such as resection was formerly suggested as the first therapeutic strategy for pediatric cases with stenosis [7, 8]. It has been recently reported that endoscopic balloon dilatation (EBD) can be a useful nonsurgical approach [10]. The optimal frequency and time of such procedures is not well established and is largely individualized. EBD has many advantages: the stricture and the esophageal mucosa can be visualized directly; the balloon catheter can be inserted to assess the effectiveness of dilatation; the degree of esophageal laceration or bleeding can be evaluated; and exposure to radiation is avoided. The common complications of EBD include esophageal perforation, recurrent stenosis, bleeding, sepsis, and mediastinitis [11–14].

## Conclusion

Intermittent vomiting, dysphagia and refusal of solid foods starting after weaning, suggest gastrointestinal conditions such as CES and not simply gastroesophageal reflux. The correct diagnosis requires radiologic and endoscopic investigations.

In our experience, as well as that of others, for congenital esophageal stenosis endoscopic balloon dilatation is the primary treatment with high percentage of clinical success.

In conclusion, CES is a rare condition with respect to gastroesophageal reflux and should be suspected when a mother reports her infant has difficulty swallowing solid foods.

Careful assessment with endoscopy is needed to diagnose CES early and referral to a pediatric gastroenterologic unit may be required.

## Abbreviations

EFI: Esophageal food impaction
CES: Congenital esophageal stenosis
EBD: Endoscopic balloon dilatation
